# Mental Health and Resilience in Nursing Students: A Longitudinal Study

**DOI:** 10.3390/ijerph22050735

**Published:** 2025-05-06

**Authors:** William Donegá Martinez, Tiago Casaleiro, João Daniel de Souza Menezes, Matheus Querino da Silva, Emerson Roberto dos Santos, Rauer Ferreira Franco, Alex Bertolazzo Quiterio, Thales Guardia de Barros, Ana Julia de Deus Silva, Luiz Otávio Maciel Lopes, Sônia Maria Maciel Lopes, Natalia Almeida de Arnaldo Silva Rodriguez Castro, Camila Aline Lázaro, Maria Laura Fabris, Josimerci Ittavo Lamana Faria, Fernando Nestor Facio Júnior, Maria Helena Pinto, Daniele Alcalá Pompeo, Denise Cristina Móz Vaz Oliani, Antônio Hélio Oliani, Neuza Alves Bonifácio, Loiane Letícia dos Santos, Marco Antonio Ribeiro Filho, Gerardo Maria de Araújo Filho, Nádia Antônia Aparecida Poletti, Luís Cesar Fava Spessoto, Natália Sperli Geraldes Marin dos Santos Sasaki, Aparecida de Fátima Michelin, Sabrina Ramires Sakamoto, Maysa Alahmar Bianchin, Vânia Maria Sabadoto Brienze, Alba Regina de Abreu Lima, Rita de Cássia Helú Mendonça Ribeiro, Júlio César André

**Affiliations:** 1 Center for Studies and Development of Health Education—CEDES, São José do Rio Preto Medical School—FAMERP, São José do Rio Preto 15090-000, Brazil; joao.menezes@edu.famerp.br (J.D.d.S.M.); matheusquirino@hotmail.com (M.Q.d.S.); emerson.santos@edu.famerp.br (E.R.d.S.); alex.quiterio@edu.famerp.br (A.B.Q.); thales.barros@edu.famerp.br (T.G.d.B.); anajuliadds@yahoo.com.br (A.J.d.D.S.); sonia.lopes@edu.famerp.br (S.M.M.L.); natalia.castro@edu.famerp.br (N.A.d.A.S.R.C.); marialaurafabris@outlook.com (M.L.F.); fnfacio@yahoo.com.br (F.N.F.J.); loiane.psicologia@gmail.com (L.L.d.S.); marcoribeirofilho@gmail.com (M.A.R.F.); maysa@famerp.br (M.A.B.); vania.brienze@hospitaldebase.com.br (V.M.S.B.); alba.lima09@gmail.com (A.R.d.A.L.); julio.andre@edu.famerp.br (J.C.A.); 2 Grupo Autónoma - Escola Superior de Enfermagem São Francisco das Misericórdias, Green Park Campus, 1600-300 Lisbon, Portugal; tcasaleiro@esesfm.pt; 3 University Brasil—UB, Fernandópolis 15613-899, Brazil; rauerf@hotmail.com; 4 Santa Casa de Misericórdia de Votuporanga, Votuporanga 15500-003, Brazil; luiz_otavio.2@hotmail.com; 5 University União das Faculdades dos Grandes Lagos—UNILAGO, São José do Rio Preto 15070-110, Brazil; camilaalazaro@gmail.com; 6 Faculty São José do Rio Preto Medical School—FAMERP, São José do Rio Preto 15090-000, Brazil; josimerci.faria@edu.famerp.br (J.I.L.F.); mariahelena@famerp.br (M.H.P.); daniele.pompeo@famerp.br (D.A.P.); vaz.oliani@gmail.com (D.C.M.V.O.); aholiani@gmail.com (A.H.O.); filho.gerardo@gmail.com (G.M.d.A.F.); nadia@famerp.br (N.A.A.P.); lcspessoto@gmail.com (L.C.F.S.); natalia.geraldes@famerp.br (N.S.G.M.d.S.S.); ritadecassia@famerp.br (R.d.C.H.M.R.); 7 University Hospital Center Cova da Beira, University of Beira Interior, 6201-001 Covilhã, Portugal; 8 Campus Araçatuba, University Paulista—UNIP, Araçatuba 16018-555, Brazil; neuzabonifacio@gmail.com (N.A.B.); cidinhamichelin@gmail.com (A.d.F.M.); 9 Faculty of Veterinary Medicine, Universidade Estadual Paulista Júlio de Mesquita Filho, Araçatuba 16050-680, Brazil; 10 Penápolis School of Philosophy, Sciences and Letters, Penápolis Educational Foundation, Penápolis 16300-000, Brazil; ramiressabrina@hotmail.com

**Keywords:** psychological resilience, nursing students, mental health, nursing education, psychological adaptation, young adults, health promotion

## Abstract

Mental health challenges are increasingly prevalent among young individuals, particularly within high-stress academic environments such as nursing education. Resilience is critical for maintaining well-being and adapting to university demands. Objective: To assess resilience levels in first-year nursing students at FAMERP (Faculty of Medicine of São José do Rio Preto) upon entry in 2021 and their longitudinal evolution in 2022 and 2023, using the Wagnild and Young Resilience Scale. The study also aims to explore the implications for mental health promotion in young healthcare professionals. Methods: A descriptive, longitudinal, prospective, and quantitative study was conducted with 40 students. Data collection was performed via electronic forms and analyzed using descriptive statistics and specific tests within the R programming language. Results: The predominantly female sample, with a mean age of 19.5 years, exhibited moderate to high resilience levels: mean scores of 132.5 (2021), 135.8 (2022), and 139.2 (2023). A significant reduction in the Perseverance factor (*p* = 0.0131) was noted. There was a positive correlation between age and resilience scores (r = 0.42; *p* < 0.01). Discussion: Despite a slight overall increase in resilience, the decline in Perseverance is concerning, indicating potential growing mental health challenges as students progress. Small age differences significantly influence mental health outcomes. This decline may be related to increased academic stress, exposure to emotionally challenging clinical situations, and the cumulative effects of the “costs of caring”. Conclusions: Nursing students exhibit promising resilience levels, yet there is a critical need for interventions targeting Perseverance to enhance academic performance and patient care quality. Social Impact: This study contributes to the development of educational strategies designed to promote resilience, thereby potentially improving the mental health and academic performance of nursing students. By focusing on mental well-being, a more resilient healthcare workforce can be cultivated and better prepared to meet systemic challenges.

## 1. Introduction

The escalating prevalence of mental health challenges among young people has become a significant concern, particularly within high-pressure academic environments such as nursing education [1,2,3,4]. Nursing education, in general, is known for its demanding curriculum, high workloads, complex decision-making, and emotional challenges, placing nursing students at a high risk of experiencing burnout, anxiety, and emotional exhaustion [5,6]. These factors can significantly impact the well-being of nursing students, including their personal relationships and future professional practice and judgment.

Resilience, defined as the ability to recover, adapt, and thrive in the face of adversity, is a crucial factor in maintaining mental well-being and adapting to the multifaceted demands of university life [5,6,7]. It encompasses effective coping mechanisms, emotional regulation, and the capacity to “bounce back” from adversity [7,8,9]. To enhance our understanding of resilience, it is useful to consider theories such as Bandura’s Self-Efficacy Theory, which highlights the importance of belief in one’s ability to execute tasks and achieve goals, and Duckworth’s theory of “Grit”, which emphasizes perseverance and passion for long-term goals [10,11,12]. Given the increasing rates of moral distress (MD), compassion fatigue (CF), vicarious trauma (VT), secondary traumatic stress (STS) and burnout among healthcare professionals, resilience is increasingly recognized as an essential attribute for those entering the nursing field [13].

This study focuses on nursing students who face unique stressors in their academic and clinical training, potentially impacting their mental health and future professional performance [14]. Nursing students are frequently exposed to emotionally challenging situations, including patient suffering, death, and ethical dilemmas, which can contribute to increased stress levels and decreased mental well-being [14,15,16,17]. These stressors can be categorized into three primary themes: (a) Academic Stress: includes the pressure to maintain good academic performance, time management, and information overload [18,19]. (b) Clinical Exposure refers to direct contact with patient suffering, death, and ethical dilemmas, which can lead to vicarious trauma and compassion fatigue [20,21]. (c) Personal and Interpersonal Challenges: encompasses the need to balance academic life with personal responsibilities, financial difficulties, and relationship issues [22,23].

To counter the effects of the costs of caring in nursing schools, efforts to cultivate resilience have been increasing [20,21]. It is hoped that training in mindfulness, self-care, time management, managing stress, developing appropriate coping strategies, and maintaining emotional regulation will bestow greater adaptability, perseverance and tolerance for adversity [22,23,24,25,26,27,28,29].

The development of resilience in nursing students is important to aid them in coping with the many stressors they face [5,6]. Resilience has been shown to positively correlate with academic performance, reduced burnout rates, and enhanced patient care quality among nursing students [30,31]. Therefore, fostering resilience is crucial for cultivating competent and compassionate healthcare professionals. However, efforts to incorporate resilience building in nursing curricula remain inconsistent due to an inadequate understanding of the requisite processes [5,6].

This research contributes to the growing body of literature focused on understanding and promoting mental health and well-being among young people, particularly those in demanding educational pathways. By investigating the resilience of nursing students, this study addresses a critical need for evidence-based strategies to support the mental health of future healthcare professionals during their formative years, ultimately aiming to enhance their capacity to provide high-quality care.

Therefore, this study aims to assess resilience levels in first-year nursing students at FAMERP (Faculty of Medicine of São José do Rio Preto) upon entry in 2021 and their longitudinal evolution in 2022 and 2023, using the Wagnild and Young Resilience Scale. By tracking changes in resilience over time, this research seeks to explore the implications for mental health promotion in young nursing professionals and contribute to the development of targeted interventions to support their well-being.

## 2. Materials and Methods

This study employed a descriptive, longitudinal, prospective, and quantitative design to assess the resilience levels of first-year nursing students at the Faculdade de Medicina de São José do Rio Preto (FAMERP) and to evaluate the changes in these levels over three academic years. The study was conducted from 2021 to 2023.

### 2.1. Participants

The study population comprised all first-year nursing students enrolled at FAMERP in 2021. A total of 40 students participated in the study, representing 6.67% of the total first-year nursing student population (*n* = 60) at FAMERP. This high participation rate is a strength of the study, especially considering the challenges inherent in longitudinal research, such as participant attrition and the commitment required for repeated data collection over three years [32]. While this represents the entire cohort, it is important to acknowledge that smaller sample sizes may have reduced statistical power to detect significant differences or associations [33]. All participants were reassessed in 2022 and 2023 to track longitudinal changes in resilience. Inclusion criteria were: (1) being a first-year nursing student at FAMERP in 2021 and (2) providing informed consent to participate in the study. There were no specific exclusion criteria.

### 2.2. Data Collection Instruments

Data were collected using an electronic form (Google Forms) that included the following components:

I. Term of Consent: An electronic Term of Consent (TC) was presented at the beginning of the form, ensuring that all participants were informed about the study’s objectives, procedures, potential risks and benefits, and their right to withdraw at any time without penalty.
II. Sociodemographic and Professional Variables Questionnaire (SDVQ): This questionnaire, developed by the researchers, collected data on participants’ demographic characteristics (e.g., age, gender, marital status, socioeconomic status) and academic background (e.g., prior education, type of high school, previous higher education experience).
III. Wagnild and Young Resilience Scale: The Brazilian version of the Wagnild and Young Resilience Scale was used to measure resilience levels [34,35]. This scale consists of 25 items, each rated on a 7-point Likert scale ranging from 1 (strongly disagree) to 7 (strongly agree). The scale assesses five dimensions of resilience [34]:
  - Self-Sufficiency: Reflects a belief in one’s own competence and independence. This factor is assessed by items 4, 6, 11, 15, and 21, with possible scores ranging from 5 to 35.
  - Meaning of Life: Indicates a sense of purpose and satisfaction with life. This factor is assessed by items 4, 6, 11, 15, and 21, with possible scores ranging from 5 to 35.
  - Equanimity: Represents the ability to remain balanced and composed in the face of adversity. This factor is assessed by items 7, 12, 16, 19, and 22, with possible scores ranging from 5 to 35.
  - Perseverance: Measures the capacity to persist in the face of obstacles and challenges. This factor is assessed by items 1, 10, 14, 20, and 24, with possible scores ranging from 5 to 35.
  - Existential Singularity: Reflects a sense of connection to something larger than oneself and acceptance of life’s uncertainties. This factor is assessed by items 3, 5, 8, 17, and 25, with possible scores ranging from 5 to 35.

Total scores range from 25 to 175, with higher scores indicating greater resilience. The Brazilian version of the Wagnild and Young Resilience Scale has demonstrated good validity and reliability in previous studies, making it a suitable instrument for assessing resilience in this population [35]. Specifically, Cronbach’s alpha for the scale was reported as 0.85, indicating high internal consistency. It is important to note that the resilience subscale scores were analyzed using raw totals, as this approach is consistent with the scale’s original guidelines and allows for a straightforward interpretation of the results [34].

### 2.3. Data Collection Procedures

The electronic form was distributed to eligible participants via email at the time of their enrollment in 2021 and during subsequent re-enrollment periods in 2022 and 2023. The email included a brief explanation of the study and a link to access the form. Participants were given ample time to complete the form and were assured of the confidentiality of their responses. Several measures were implemented to ensure data quality. Data were checked for internal consistency and outliers, and participants were contacted to clarify any inconsistencies or missing information. In addition, data entry was double-checked to minimize errors. During the COVID-19 pandemic (2021 and 2022), data collection was conducted entirely online due to restrictions on in-person activities.

### 2.4. Data Analysis

Data were analyzed using the R programming language (version 4.0.3) and the following packages: “dplyr”, “ggplot2”, “rstatix”, and “boot” [36]. The reliability of the instrument with the sample was assessed using Cronbach’s alpha, which was found to be 0.85 for the entire scale, and the following values for each factor: Self-Sufficiency (α = 0.75), Meaning of Life (α = 0.80), Equanimity (α = 0.72), Perseverance (α = 0.78), and Existential Singularity (α = 0.70). Descriptive statistics, including means, standard deviations, frequencies, and percentages, were calculated to summarize the sociodemographic characteristics of the participants and their resilience scores. The Shapiro-Wilk test was used to assess the normality of data distribution. Repeated measures ANOVA was used to compare resilience scores across the three time points (2021, 2022, and 2023). Post-hoc tests (Bonferroni correction) were performed to identify specific time points where significant differences occurred. Effect sizes (partial eta squared) were calculated to determine the magnitude of the observed differences. Due to the non-normal distribution of residuals for the “Meaning of Life” factor, a bootstrapping technique was applied to estimate a more robust *p*-value. Bootstrapping is a resampling method that involves repeatedly drawing random samples with replacements from the original data to create multiple simulated datasets [37]. This allows for the estimation of standard errors and confidence intervals without relying on assumptions of normality. In this case, we used bootstrapping to generate 1000 resamples and calculate a more accurate *p*-value for the “Meaning of Life” factor. This approach provides a more reliable assessment of statistical significance when the assumption of normality is violated [38]. Statistical significance was set at *p* < 0.05.

### 2.5. Ethical Considerations

This study was conducted in accordance with the ethical principles outlined in the Declaration of Helsinki. The research protocol was reviewed and approved by the Institutional Ethics Committee of FAMERP (protocol code 4.543.158, 17 February 2021). All participants provided informed consent prior to their participation in the study. Participants were informed of their right to withdraw from the study at any time without penalty. All data were anonymized to protect the privacy of the participants. In addition to these standard ethical procedures, it is important to note that while the study itself did not provide direct mental health support, participants were informed about the availability of mental health services at FAMERP and were encouraged to seek assistance if they experienced elevated distress during the study. Information on how to access these services was provided to all participants, along with the informed consent form.

## 3. Results

The sample comprised 40 nursing students from FAMERP, predominantly female (85%), with a mean age of 19.5 years (SD = 1.8). Table 1 presents the descriptive statistics for the resilience dimensions across the three years of the study, revealing moderate to high resilience levels among the participants.

Table 1 presents the descriptive statistics for resilience factors, indicating moderate to high levels of resilience among students, particularly in Self-Reliance and Perseverance.

To understand how the resilience factors changed over time, a repeated measures ANOVA was conducted. Table 2 summarizes the results of this analysis, highlighting the statistical significance and effect sizes for each factor.

Given the significant finding for the Perseverance factor, post-hoc tests were performed to pinpoint where the differences occurred. Table 3 details these comparisons between the years, providing adjusted *p*-values and Cohen’s d effect sizes.

While the decrease in Perseverance was statistically significant, it is important to consider the practical implications of this change. A decrease of 2.55 points on the scale may indicate a reduced capacity to cope with academic stress and pressure, potentially affecting performance and well-being.

**Figure 1:**
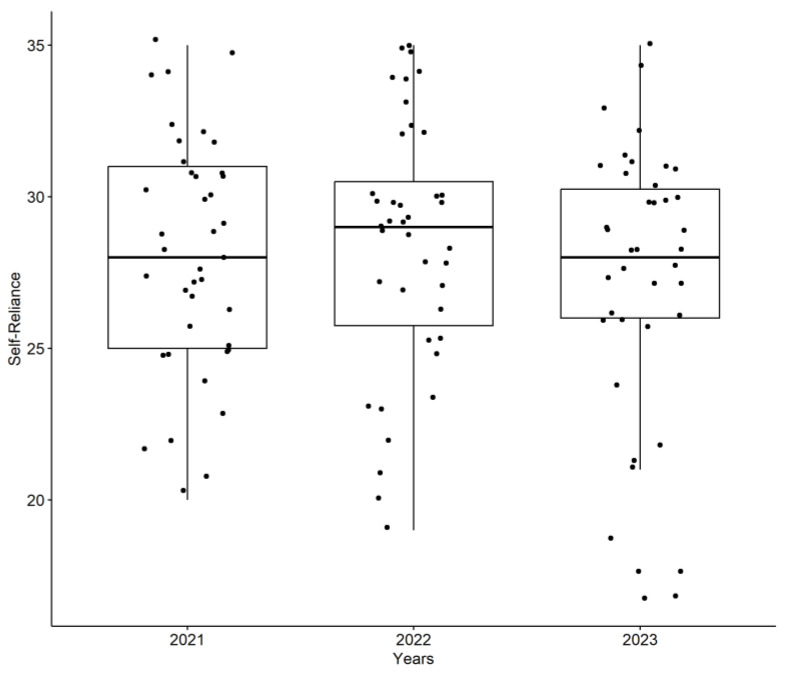
Self-Reliance scores across the three years of the study (2021–2023) demonstrate the stability of this resilience factor. (*n* = 40, FAMERP, 2025).

To illustrate the trend in Self-Sufficiency, which remained relatively stable over the study period, Figure 1 presents the scores for this factor across the three years.

**Figure 2:**
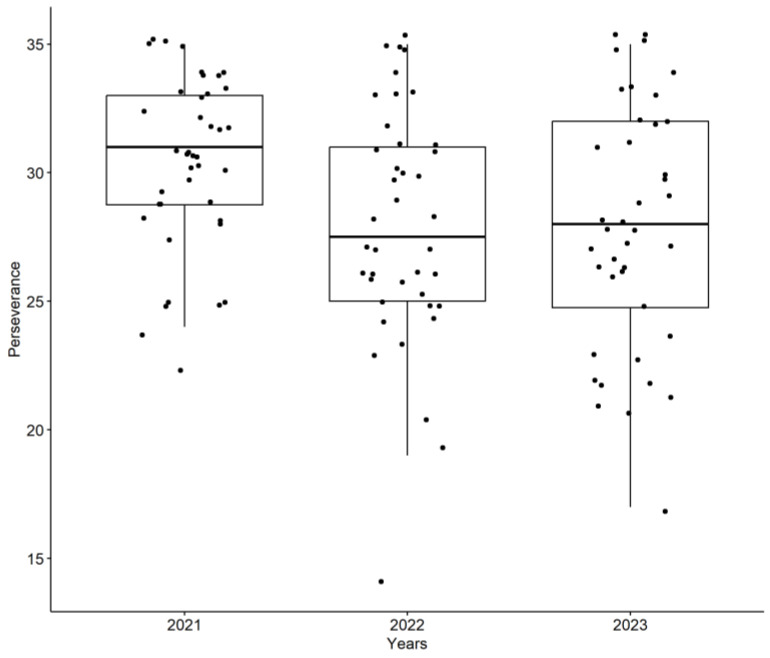
Perseverance scores across the three years of the study (2021–2023) illustrate the significant decrease in this factor. (*n* = 40, FAMERP, 2025).

In contrast to Self-Reliance, the Perseverance factor showed a significant decline. To further emphasize the importance of this finding, we have added a more detailed explanation of the observed decrease in Perseverance scores. Specifically, the Perseverance scores decreased significantly from 2021 (M = 30.40, SD = 3.37) to 2022 (M = 27.95, SD = 4.70) and 2023 (M = 27.85, SD = 4.68), as revealed by repeated measures ANOVA (*p* = 0.0131). Post-hoc comparisons indicated significant differences between 2021 and 2022 (*p* = 0.027) and between 2021 and 2023 (*p* = 0.021). Figure 2 visually represents this decrease in Perseverance scores from 2021 to 2022 and 2023, highlighting the impact of academic progression on this aspect of resilience.

**Figure 3:**
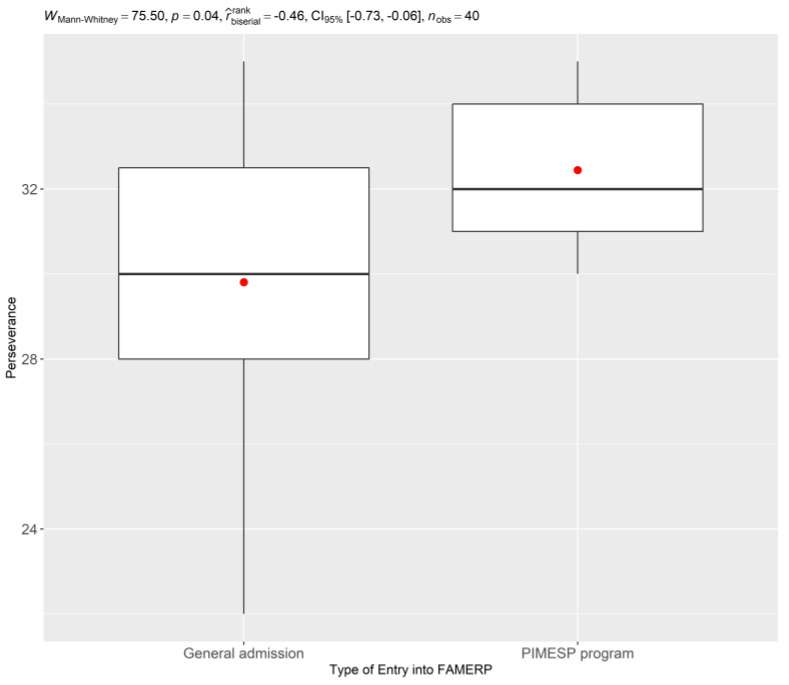
Comparison of Self-Reliance by Sex, indicating no significant differences between groups. (*n* = 40, FAMERP, 2025).

To explore whether sociodemographic factors influenced resilience, comparisons were made between different groups. Figure 3 presents the comparison of A Self-Reliance by sex, showing that there were no significant differences between male and female students in this resilience factor.

**Figure 4:**
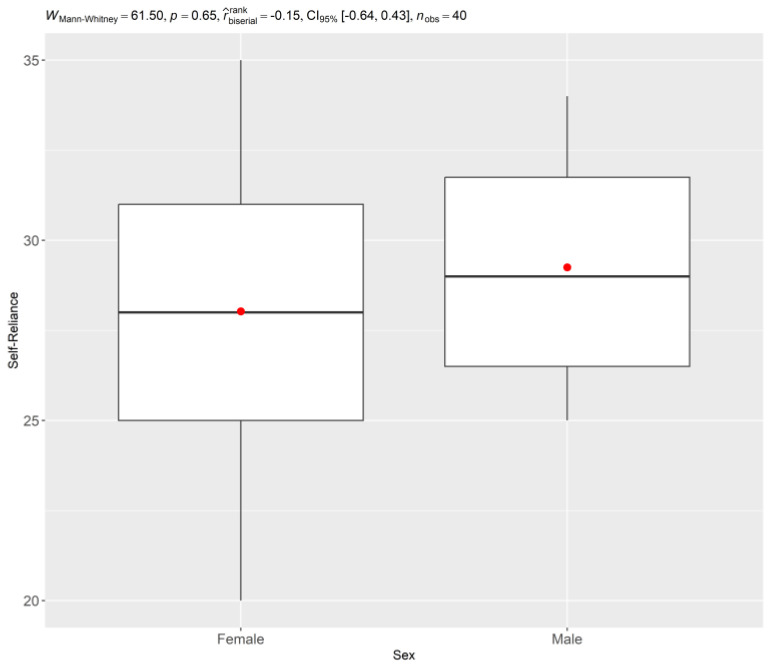
Comparison of Perseverance by Type of Entry into FAMERP, demonstrating higher scores for students entering through the PIMESP program (Inclusion Program with Merit in Public Higher Education of São Paulo State). (*n* = 40, FAMERP, 2025).

Finally, Figure 4 presents a comparison of Perseverance scores based on the type of entry into FAMERP, revealing that students entering through the PIMESP program (Inclusion Program with Merit in Public Higher Education of São Paulo State) exhibited higher scores in Perseverance compared to those entering through general admission.

Independent samples t-tests were conducted to determine if there were statistically significant differences in Perseverance scores based on sex and type of entry into FAMERP. The *t*-tests comparing Perseverance scores by sex were not significant for any of the time points (2021, 2022, and 2023). However, the t-test comparing Perseverance scores by type of entry into FAMERP revealed a significant difference in 2021 (t = 2.5, *p* < 0.05), with students entering through the PIMESP program exhibiting higher Perseverance scores. This difference was not significant in 2022 or 2023.

These results indicate that while nursing students at FAMERP begin their course with promising resilience levels, there is a notable decline in the Perseverance factor over time. Additionally, the type of entry into the university appears to influence students’ perseverance levels.

## 4. Discussion

This longitudinal study aimed to assess resilience levels in first-year nursing students at FAMERP upon entry in 2021 and to track their evolution over three academic years. The findings reveal a complex interplay between resilience and mental health in this population, underscoring the need for targeted interventions to support their well-being throughout their demanding educational journey.

The initial assessment revealed moderate to high resilience levels among the nursing students, suggesting a solid foundation for navigating the inherent challenges of their chosen field. This finding aligns with previous research emphasizing resilience as a protective factor against stress and burnout in healthcare professionals [7,8,9,39,40,41]. However, it is important to note that some studies have reported lower resilience levels in similar populations, potentially attributable to variations in sample characteristics or measurement instruments [40,42].

However, the longitudinal analysis revealed a significant decrease in the Perseverance factor over the three-year period. This decline is concerning and may indicate a growing vulnerability to mental health challenges as students progress through their rigorous program, including increased workload demands, exposure to emotionally challenging clinical situations, and the ongoing impact of the COVID-19 pandemic, which has been associated with increased stress and mental health issues among healthcare students [43]. The decrease in perseverance could be attributed to a multitude of factors, including increased academic stress, exposure to emotionally demanding clinical situations, and the cumulative effects of the “costs of caring” [13]. While the statistical significance of the decline in Perseverance is clear (*p* = 0.0131), it is important to consider the clinical relevance of this change. A decrease of 2.55 points on the scale, while statistically significant, may have a meaningful impact on students’ ability to cope with the demands of their program. This decline could manifest as reduced motivation, increased difficulty persisting through challenging tasks, and a greater susceptibility to burnout [44,45]. It is important to note that the significant decrease in the Perseverance factor was primarily observed in the 2021–2022 period, with a small effect size (partial eta squared = 0.1), as indicated in Table 2. The difference in Perseverance between 2022 and 2023 was not statistically significant (Table 3).

The finding that the Perseverance factor declined over time warrants further investigation. It is possible that the initial enthusiasm and motivation that characterize students upon entering nursing school may wane as they encounter the realities of clinical practice and the demands of their coursework. This highlights the need for interventions that specifically target the maintenance of perseverance and motivation throughout the nursing program. Several factors may contribute to this decline in perseverance. These include increased clinical demands, which can lead to exhaustion and a sense of being overwhelmed; a perceived lack of institutional support, which can undermine students’ confidence and motivation; and disillusionment with the profession, as students encounter the realities of healthcare and the challenges of balancing work and personal life [46,47]. These insights, while hypothetical in the context of our study, are supported by the existing literature on the experiences of nursing students. In addition, the decline in perseverance may be related to increased academic stress throughout the course. Studies have shown that excessive workload, pressure for good performance, and information overload can lead to mental and emotional exhaustion, undermining motivation and the ability to persist in the face of challenges [48]. Furthermore, the lack of time for leisure activities and self-care can contribute to burnout and decreased perseverance. Exposure to emotionally challenging clinical situations, such as patient suffering, death, and ethical dilemmas, can have a significant impact on the perseverance of nursing students. Experiencing these situations can lead to compassion fatigue, post-traumatic stress, and burnout, affecting the ability to maintain motivation and persistence in the face of obstacles [49]. It is important to emphasize that the lack of adequate preparation and emotional support during clinical experiences can exacerbate these effects. The concept of “costs of caring” refers to the emotional and psychological impact of caring for others, especially in situations of suffering and vulnerability. In the context of nursing education, students may experience the costs of caring when faced with the pain, suffering, and death of patients, as well as when dealing with the emotional demands of their families. These costs can lead to emotional exhaustion, depersonalization, and decreased perseverance [50].

The positive correlation between age and resilience scores, while moderate, suggests that even small age differences within this young cohort (mean age 19.5 years) may influence mental health outcomes. This finding emphasizes the need for tailored mental health interventions that consider the developmental stages and experiences of young adults in healthcare education. Older students may possess greater life experience and coping skills that contribute to their resilience, while younger students may require additional support to develop these skills.

Interestingly, the study found that students entering through the PIMESP program (Inclusion Program with Merit in Public Higher Education of São Paulo State) exhibited higher scores in Perseverance compared to those entering through general admission. This suggests that students from disadvantaged backgrounds may possess unique strengths and coping mechanisms that contribute to their resilience. There may be structural or psychosocial explanations for this disparity. For example, students entering the PIMESP program may have developed greater perseverance due to the challenges they have overcome in accessing higher education. They may also have stronger social support networks or a greater sense of purpose, which could contribute to their resilience [51,52]. Further research is needed to explore the specific factors that contribute to the higher perseverance levels among PIMESP students. Further research is needed to explore the specific factors that contribute to the higher perseverance levels among PIMESP students.

Overall, the data suggest that nursing students who choose this path with conviction tend to be highly resilient and that, despite adverse circumstances, they do not give up easily. Despite the difficulties (e.g., COVID-19), they did not show major changes in the factors used to assess their level of resilience. While the Perseverance factor decreased significantly during the most challenging period of COVID-19 (2021–2022), the effect size of this variation was small (eta squared around 0.1), indicating a limited impact on their overall resilience. The other factors (self-sufficiency, meaning of life, equanimity, and existential singularity) did not diminish significantly.

These findings should be interpreted within the context of the study’s limitations. The sample size was relatively small (*n* = 40) and limited to a single institution, which may limit the generalizability of the findings. Additionally, the study relied on self-report measures, which may be subject to bias. Future research should explore the use of mixed-methods designs to gain a more in-depth understanding of the factors influencing resilience in nursing students.

The results of this study have important implications for nursing education and practice. The findings underscore the need for proactive interventions to promote mental health and well-being among nursing students. These interventions should focus on:

- Stress management techniques: Providing students with training in mindfulness, self-care, and coping strategies to manage stress and prevent burnout [22,23,24].
- Emotional regulation skills: Helping students develop the ability to identify and regulate their emotions in response to challenging clinical situations [5,6].
- Supportive learning environment: Creating a supportive and inclusive learning environment that fosters a sense of belonging and reduces stigma associated with mental health issues [5,6].
- Mentorship programs: Pairing students with experienced nurses who can provide guidance, support, and role modeling [5,6].
- Curriculum integration: Integrating resilience-building activities into the nursing curriculum to ensure that all students receive training in these essential skills [5,6].

By implementing these strategies, nursing programs can better prepare students to navigate the challenges of their profession and promote their long-term mental health and well-being.

## 5. Conclusions

This longitudinal study provides valuable insights into the resilience levels of nursing students at FAMERP, revealing both promising strengths and potential vulnerabilities throughout their academic journey. The initial assessment indicated that these students possess moderate to high levels of resilience upon entering the program, suggesting a solid foundation for navigating the challenges inherent in nursing education. However, the subsequent longitudinal analysis revealed a concerning trend: a significant decrease in the Perseverance factor over the three-year period. This decline highlights the potential impact of academic stressors, exposure to demanding clinical situations, and the cumulative effects of the “costs of caring” on this crucial aspect of resilience.

These findings underscore the complex interplay between resilience and mental health in young nursing students and emphasize the need for proactive interventions to promote their well-being. By understanding the specific challenges faced by these students, educators and institutions can develop targeted strategies to mitigate the negative effects of stress and foster a more supportive and resilient learning environment.

### 5.1. Social Impact

This study contributes to the development of educational strategies that promote resilience in nursing students, potentially improving their mental health, academic performance, and, consequently, the quality of care provided to patients in the future. By focusing on young healthcare professionals’ mental well-being, we can foster a more resilient and mentally healthy workforce that is better equipped to handle the challenges of the healthcare system. Furthermore, by identifying factors that contribute to resilience, this research can inform the design of interventions that promote well-being not only for nursing students but also for other young people in demanding educational pathways.

### 5.2. Implications for Education

The findings of this study have several important implications for nursing education. First, they highlight the need for nursing programs to proactively address mental health and well-being among students. This can be achieved by integrating resilience-building activities into the curriculum, providing training in stress management and emotional regulation techniques, and creating a supportive and inclusive learning environment. Second, the study suggests that tailored interventions may be needed to address the specific needs of younger students, who may lack the life experience and coping skills of their older peers. Finally, the study underscores the importance of considering the unique strengths and challenges of students from disadvantaged backgrounds, such as those entering through the PIMESP program and developing interventions that build on their existing resilience. To make these recommendations more concrete, we suggest the following: Resilience-building activities could be integrated into existing courses, such as clinical rotations or seminars, and could include mindfulness exercises, stress management workshops, and peer support groups. Training in emotional regulation techniques could be provided through workshops or online modules and could focus on helping students identify and manage their emotions in challenging clinical situations. Supportive learning environments could be fostered by creating opportunities for students to connect with faculty and peers and by promoting a culture of open communication and mutual respect [53,54].

### 5.3. Limitations

These findings should be interpreted within the context of the study’s limitations. The sample size was relatively small (*n* = 40) and limited to a single institution, which may limit the generalizability of the findings. To address this limitation more explicitly, we acknowledge that the findings may not be representative of all nursing students and that further research is needed to confirm these results in larger and more diverse samples [55]. Furthermore, the limited sample size may have reduced the statistical power to detect significant differences or associations, particularly for smaller effect sizes. While we observed a statistically significant decline in the Perseverance factor, the small sample size may limit the confidence in the generalizability of this finding. Similarly, the single-institution scope restricts the extent to which these findings can be applied to other nursing programs with different characteristics or student populations. Future research should aim to replicate this study with larger, multi-institutional samples to enhance the generalizability and robustness of the findings. Additionally, the study relied on self-report measures, which may be subject to bias. The cross-sectional nature of the data limits the ability to draw causal inferences. Furthermore, the box plots revealed increased variability in some resilience factors, with a few subjects exhibiting notably lower self-reliance values and one case in Perseverance in 2022 showing a significant decrease. While a formal outlier analysis was beyond the scope of this study, acknowledging this variability is important, and future research could benefit from exploring these outliers to gain a deeper understanding of the individual factors that may contribute to significant variations in resilience. Future research should explore the use of mixed-methods designs to gain a more in-depth understanding of the factors influencing resilience in nursing students. The findings of this study have important implications for nursing education. They suggest that nursing programs should prioritize the promotion of resilience among students by providing emotional support, coping skills training, and opportunities to develop a sense of purpose and meaning in their work. Furthermore, the results highlight the importance of monitoring students’ mental health over time and offering early interventions for those at risk of developing mental health problems.

The findings of this study have important implications for nursing education. They suggest that nursing programs should prioritize the promotion of resilience among students by providing emotional support, coping skills training, and opportunities to develop a sense of purpose and meaning in their work. Furthermore, the results highlight the importance of monitoring students’ mental health over time and offering early interventions for those at risk of developing mental health problems.

### 5.4. Future Perspectives

Future research should focus on developing and evaluating the effectiveness of targeted interventions to promote resilience among nursing students. These interventions should be tailored to address the specific challenges faced by students at different stages of their education and should incorporate a variety of strategies, such as mindfulness training, cognitive behavioral therapy, and mentorship programs. Future research should also employ mixed-methods designs to gain a deeper understanding of the experiences of nursing students and the factors that influence their resilience. The inclusion of a control group would allow for comparisons with students not enrolled in the nursing program. Furthermore, investigating mediating and moderating factors, such as social support, coping strategies, and self-efficacy, could provide valuable insights into the relationship between resilience and mental health. Additionally, future research should explore the use of longitudinal designs to track changes in resilience over time and to examine the long-term impact of resilience on academic performance, professional well-being, and patient care outcomes. Further studies with larger and more diverse samples are needed to confirm these findings and to explore the generalizability of the results to other populations. To build on the findings of this study, future research could employ a mixed-methods approach, combining quantitative data with qualitative interviews to gain a deeper understanding of the lived experiences of nursing students and the factors that influence their resilience trajectories. This could involve conducting in-depth interviews with students to explore their perceptions of stress, coping strategies, and sources of support. Furthermore, future research could investigate how resilience affects not only academic outcomes but also clinical competence and empathy, as these are essential qualities for nursing professionals [56].

## Figures and Tables

**Table 1:** Descriptive analysis of resilience factors over three years (2021–2023). (*n* = 40, FAMERP, 2025).

	Year		
Resilience Factor	2021	2022	2023
Self-Sufficiency	28.15 (3.85)	28.43 (4.22)	27.13 (4.72)
Meaning of Life	27.95 (2.19)	26.33 (3.42)	26.75 (2.84)
Equanimity	24.55 (4.38)	25.25 (4.62)	23.33 (4.15)
Perseverance	30.40 (3.37)	27.95 (4.70)	27.85 (4.68)
Existential Singularity	27.83 (4.45)	27.10 (4.88)	27.23 (5.20)

Results are presented as mean (standard deviation).

**Table 2:** Comparative analysis of resilience factors over three years (2021–2023). (*n* = 40, FAMERP, 2025).

	Year				
Resilience Factor	2021	2022	2023	*p*-Value *	Effect Size †
Self-Sufficiency	28.15 (3.85)	28.43 (4.22)	27.13 (4.72)	0.3857	0.0241
Meaning of Life	27.95 (2.19)	26.33 (3.42)	26.75 (2.84)	0.1200 ‡	0.0927
Equanimity	24.55 (4.38)	25.25 (4.62)	23.33 (4.15)	0.1148	0.0540
Perseverance	30.40 (3.37)	27.95 (4.70)	27.85 (4.68)	**0.0131**	0.1051
Existential Singularity	27.83 (4.45)	27.10 (4.88)	27.23 (5.20)	0.7851	0.0062

Results are presented as mean (standard deviation). * Repeated measures ANOVA; † Effect size = partial eta squared. ‡ Estimated mean *p*-value after applying bootstrapping technique due to non-normal distribution of residuals. Bold formatting = Significant *p*-value.

**Table 3:** Post-hoc comparisons for perseverance factor (2021–2023). (*n* = 40, FAMERP, 2025).

	Pairwise Comparisons					
	2021 vs. 2022		2021 vs. 2023		2022 vs. 2023	
Resilience Factor	*p*-Value	TDE (IC 95%)	*p*-Value	TDE (IC 95%)	*p*-Value	TDE (IC 95%)
Perseverance	**0.027**	0.570 0.147; 0.994	**0.021**	0.594 0.171; 1.016	1.000	0.023 −0.479; 0.525

ES, Effect Size = Cohen’s d; CI, Confidence Interval; Bold formatting = Significant *p*-value.

## Data Availability

The original contributions presented in this study are included in the article Further inquiries can be directed to the corresponding author.
